# Improving carbon sequestration estimation through accounting carbon stored in grassland soil

**DOI:** 10.1016/j.mex.2019.12.003

**Published:** 2019-12-07

**Authors:** M.F. Ricard, E.F. Viglizzo

**Affiliations:** a Instituto de Ciencias de la Tierra y Ambientales de La Pampa - Consejo Nacional de Investigaciones Científicas y Técnicas, Mendoza 109, L6300, Santa Rosa, La Pampa, Argentina; b Facultad de Ciencias Exactas y Naturales, Universidad Nacional de La Pampa, Uruguay 151 (L6300), La Pampa, Argentina; c GPS Grupo de Países Productores del Sur, Billinghurst 2565 - 4º floor (C1425DTY), Ciudad Autónoma de Buenos Aires, Argentina; d Universidad Austral, Paraguay 1950 (S2000FZF), Rosario, Santa Fe, Argentina

**Keywords:** Carbon balance in grazing lands, SOC sequestration, Grazing lands, Theoretical method, Empirical validation

## Abstract

Based on international guidelines, the elaboration of national carbon (C) budgets in many countries has tended to set aside the capacity of grazing lands to sequester C as soil organic carbon (SOC). A widely applied simple method assumes a steady state for SOC stocks in grasslands and a long-term equilibrium between annual C gains and losses. This article presents a theoretical method based on the annual conversion of belowground biomass into SOC to include the capacity of grazing-land soils to sequester C in greenhouse gases (GHG) calculations. Average figures from both methods can be combined with land-use/land-cover data to reassess the net C sequestration of the rural sector from a country. The results of said method were validated with empirical values based on peer-reviewed literature that provided annual data on SOC sequestration.

This methodology offers important differences over pre-existing GHG landscape approach calculation methods:

• improves the estimation about the capacity of grazing-land soils to sequester C assuming these lands are not in a steady state and
• counts C gains when considering that grazing lands are managed at low livestock densities.

## Graphical abstract

**Figure:**
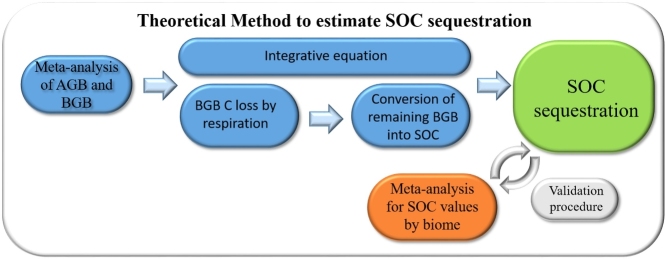

**Specification Table**

**Table:** 

Subject Area:	Environmental Science
More specific subject area:	Agro-ecosystems
Method name:	Carbon balance in grazing lands
Name and reference of original method:	IPCC Methodological Guidelines (Source: IPCC. 2006. Guidelines for National Greenhouse Gas Inventories Volume 4: Agriculture, Forestry, and Other Landuse. OECD Press, Paris (2006) 505 p.)
Resource availability:	Databases: HYDE 3.1 global database https://themasites.pbl.nl/tridion/en/themasites/hyde/download/index-2.html EDGAR v4.2 https://edgar.jrc.ec.europa.eu/overview.php?v=42 EDGAR v4.2 FT2010 https://edgar.jrc.ec.europa.eu/overview.php?v=42FT2010

## Method details

### Method overview

Extensively used international guidelines such as those of Intergovernmental Panel on Climate Change (IPCC) Tier 1 (1996, 2006) provide thorough procedures to estimate national carbon (C) emissions. Nevertheless, methods to assess the capacity of plants and soils to capture and store C raise uncertainty because these set aside the capacity of grazing lands to sequester C as soil organic carbon (SOC). National communication reports on greenhouse gases (GHG) that applied IPCC Tier 1 procedure [1], have generally assumed that C gains and losses in grasslands are in equilibrium with a net zero C balance. Relying on a broad corpus of evidence, it is possible to insure that SOC in grazing lands are far from equilibrium and tend to gain more carbon than they lose unless the C stock reaches an uncertain saturation point. This methodology propose an alternative estimation derived from the meta-analysis of science-based, peer-reviewed data that allows to calculate the capacity of grazing lands to sequester C in soil. Then, we proposed a novel method to estimate SOC sequestration of grazing lands that are managed at low livestock densities. This theoretical method was applied to estimate SOC sequestration in the rural lands of four countries (Argentina, Brazil, Paraguay and Uruguay) in the so-called MERCOSUR (Mercado Común del Sur) due to its relevant role in the global food security [2] and its multiple climatic regions [3]. This methodology did not rely on changes of C stock over time. Instead, C sequestration was estimated on annual basis based on a new equation developed to estimate SOC using as input information from global grilled databases. Following this method, we allocated average values of C sequestration per year to different typologies of land use and land cover in different climatic regions.

**Fig. 1:**
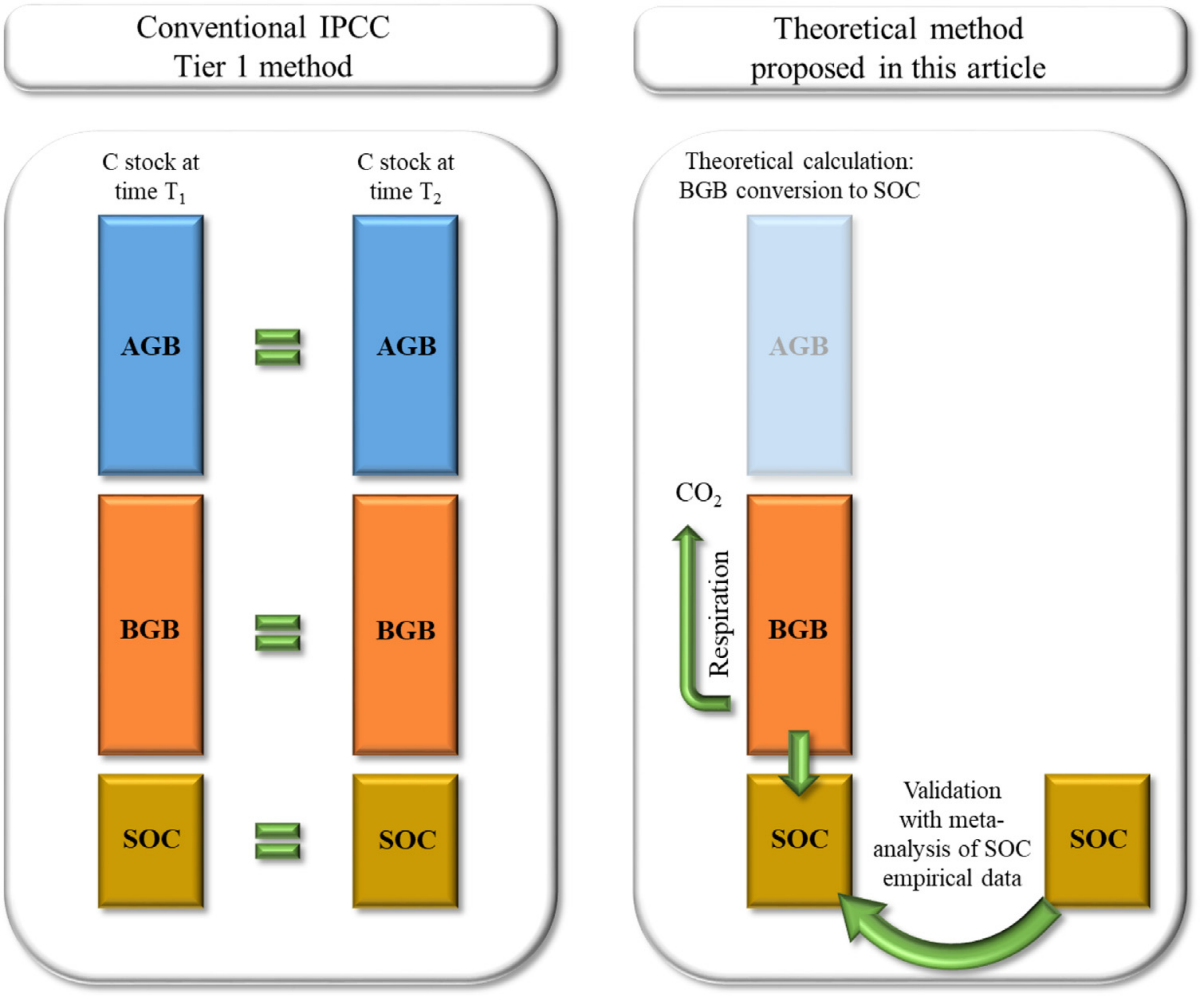
Simplified scheme showing the difference between two methods (IPCC Tier 1 and the theoretical method in this article) to estimate C sequestration in grazing lands. References: AGB (aboveground biomass), BGB (belowground biomass), SOC (soil organic carbon).

In Fig. 1, we present a simplified scheme that allows understanding the difference between the IPCC Tier 1 method (internationally incorporated for the elaboration of national greenhouse gas inventories), and the theoretical method presented in this article. To estimate annual C gain or loss, this analysis was centered on rate of SOC change in a given year, and not on the long-term change of C stocks in biomass and soil as IPCC Tier 1 does. Our method was centered on above ground biomass (AGB) and below ground biomass (BGB) relations in different biomes and climatic regions. BGB was chosen as a theoretical route to indirectly estimate SOC change, assuming that a significant proportion of C in BGB is incorporated as stable SOC once respiratory C losses are discounted. Results obtained with this method were validated with empirical data on SOC from meta-analysis of peer-reviewed literature. Gathered data were grouped for different biomes and climatic regions. A simple regression analysis was used for the validation process. We assumed that if theoretical results and empirical data were highly correlated, BGB-C could be useful to estimate sequestration as SOC.

#### Databases for land use and Carbon emission from rural lands

Data on land-use/land-cover by biome types was provided by the HYDE 3.1 global database [3]. HYDE (History Database of the Global Environment) 3.1, which is a tool that provides long-term global data on land-use/land-cover change in different biomes from 1700 to present on a grid resolution of 0.5 degress longitude/latitude. The analyzed biomes included forests, shrublands, grasslands, savannas, desert steppes, cultivated pastures and croplands distributed across four climatic regions: tropical, subtropical, temperate and cold throughout a gradient from humid to dry.

Annual data on GHG emissions (E) due to land-use change and livestock/crop production was provided by the global database EDGAR (Emission Database for Global Atmospheric Research) 4.2 [4] for the period 1970–1990, and from EDGAR v4.2 FT2010 for the year 2010. GHG emissions from rural sector (enteric fermentation, manure management, agricultural soils, indirect N_2_O emissions from agriculture, agricultural waste burning and large scale biomass burning) for each MERCOSUR country was allocated on a spatial 0.1° x 0.1° grid. Because all calculations were expressed in terms of C, a factor = 0.273 (IPCC 2006/ [1]) was used to convert GHG emissions (CO_2_ eq year^−1^) into C emissions (ton C year^−1^).

#### Proposed method: Theoretical assessment of SOC sequestration

The theoretical estimation of the annual C balance (CB) was the result of the difference between carbon emissions (E) and soil organic carbon (SOC) change (S_SOC_), which reflected an annual gain, loss or equilibrium in SOC figures.

CB = S_SOC_ – E

On the other hand, S*_SOC_* was calculated by of summing the annual contribution of each biome to SOC. The following equation summarizes the calculation procedure for each spatial unit:

$${\text{S}}_{SOC}=\sum_{B=1}^{n}(AGBg\text{*}\frac{BGB}{AGB}\text{*}0.47)-L$$

Where *B* represents the biome type with n = 6 (forest, shrublands, grasslands and savannas, steppe and sparse vegetation, cultivated pastures and croplands); *AGB_g_* is the annual growth of aboveground biomass expressed in ton dry matter (DM) ha^−1^ year^−1^; *BGB/AGB* is the relationship of each specific biome type; 0.47 is the carbon factor used to convert DM biomass into C biomass suggested by IPCC [1], and *L* represents the loss of carbon from BGB due to respiration under different thermal conditions before being converted into SOC [[5], [6], [7]].

The annual SOC sequestration was estimated following a sequence of three steps. The first one consisted of determining, for each biome and climatic region, the stock of AGB expressed in tons DM ha^−1^. DM estimations for natural forests, forest plantations, shrublands, savannas, grasslands, cultivated pastures, desert steppes and croplands were provided by a global database that comprised 685 geographical sites [8]. The second step aim at estimating BGB DM and annual BGB DM growth through BGB-AGB relations compiled for different woody and non-woody biomes across different climatic regions. Such relations result from the compilation of 402 results of different studies (See Table S1). Data on forests and shrublands were provided by IPCC guidelines [1]. Data on the annual C input from BGB in woody and non woody biomes (savannas, grasslands, pastures and annual crops) were provide by various studies [[9], [10], [11], [12], [13], [14], [15], [16], [17], [18], [19], [20], [21], [22], [23], [24], [25], [26], [27], [28], [29]]. The third step consist of calculating the proportion of the annual C input that BGB derives to SOC. To do that, calculations were based on average values in a meta-analysis work that comprised 190 results from studies of Gill and Jackson [5] and other authors [30]. Thus, respiration losses from BGB were respectively estimated in 10%, 34%, 53% and 56% for (i) forests, (ii) shrublands, (iii) grasslands, savannas, cultivated pastures, and (iv) croplands. An algorithm was also developed to include additional losses due to the average thermal conditions of each climate region, where the maximum loss occurs in the warmest regions and the minimum in the coldest ones [5]. Data on forests and shrublands was provided by IPCC guidelines 2006 [1]. The annual C input from BGB in woody and non woody biomes (savannas, grasslands, pastures and annual crops) was also estimated from 402 studies reported by IPCC 2006 [1] based on previous studies.

#### Method validation: Empirical assessment of SOC sequestration

To validate results from theoretical method, annual data on SOC from various published peer-reviewed independent studies of dominant biomes and regions [[31], [32], [33], [34], [35], [36], [37], [38], [39], [40], [41], [42], [43], [44], [45]] was used. These biomes include cultivated pastures, grasslands and savannas [31,32,34,35,39,[45], [46], [47], [48]], and croplands [34,[48], [49], [50]]. A meta-analysis was applied to synthesize information Regarding the original compilation, we decided to discard data that raised uncertainty by adopting the following criteria: i) data with incomplete information about the investigation, ii) incomplete reporting of missing data, iii) incomplete information of essential processes (e.g., ecological transitions), iii) small sampling size; iv) mean value and standard deviation in data that were extremely high or low in relation to the mean and standard deviation of the whole database, v) data that did not reflect the current condition of the local environment (for example, results from experiments involving a CO_2_ enriched atmosphere). The final database cases from 366 peer-reviewed publications for different climatic regions (See Table S2).

These collected data were counteracted with the results obtained with the theoretical method. The objective was to submit it to a validation process to verify its strength. For this, a simple linear regression model was used. The statistical significance of the model and the values of the coefficients of determination and correlation, together with the behaviour of the residuals, were verified. The high and significant relationship between the results coming from the proposed theoretical method with the empirical data that emerged from meta-analysis, gave it additional strength when estimating soil carbon sequestration. The relevance of this method is that it allows obtaining different carbon balance results when incorporating carbon sequestration in grazing lands into calculations. Examples of estimation and results obtained with this methodology compared with IPCC Tier 1 results can be seen in Viglizzo et al. [51].
